# Supplementary material for: BNST GABAergic neurons modulate wakefulness over sleep and anesthesia
Source: Commun Biol. 2024 Mar 19;7:339. doi: 10.1038/s42003-024-06028-5 (PMC10950862; doi:10.1038/s42003-024-06028-5)
Supplement: Supplementary file 2 — Supplementary Information [file 42003_2024_6028_MOESM2_ESM.pdf]

Supplementary Information

BNST GABAergic neurons modulate wakefulness over sleep and anesthesia

Mengyao Li[1,#], Wen Li[2,3,#], Shanshan Liang[3], Xiang Liao[4], Miaoqing Gu[1], Huiming Li[5], Xiaowei Chen[1,6], Hongliang Liu[7,*], Han Qin[6,*], Jingyu Xiao[7,*]

[1]Advanced Institute for Brain and Intelligence, School of Medicine, Guangxi University, Nanning 530004, China

[2]Department of Neurology, Daping Hospital, Third Military Medical University, Chongqing 400042, China

[3]Brain Research Center and State Key Laboratory of Trauma, Burns, and Combined Injury, Third Military Medical University, Chongqing 400038, China

[4]Center for Neurointelligence, School of Medicine, Chongqing University, Chongqing 400044, China

[5]Department of Anesthesiology and Perioperative Medicine, Xijing Hospital, Fourth Military Medical University, Xi'an 710032, Shaanxi, China

[6]Chongqing Institute for Brain and Intelligence, Guangyang Bay Laboratory, Chongqing 400064, China

[7]Department of Anesthesiology, Chongqing University Cancer Hospital, Chongqing 400030, China

[#]These authors contributed equally to this work

*Corresponding authors:

liuhl75@163.com (HL); qinhan66@foxmail.com (HQ); jyxiao1989@cqu.edu.cn (JX)

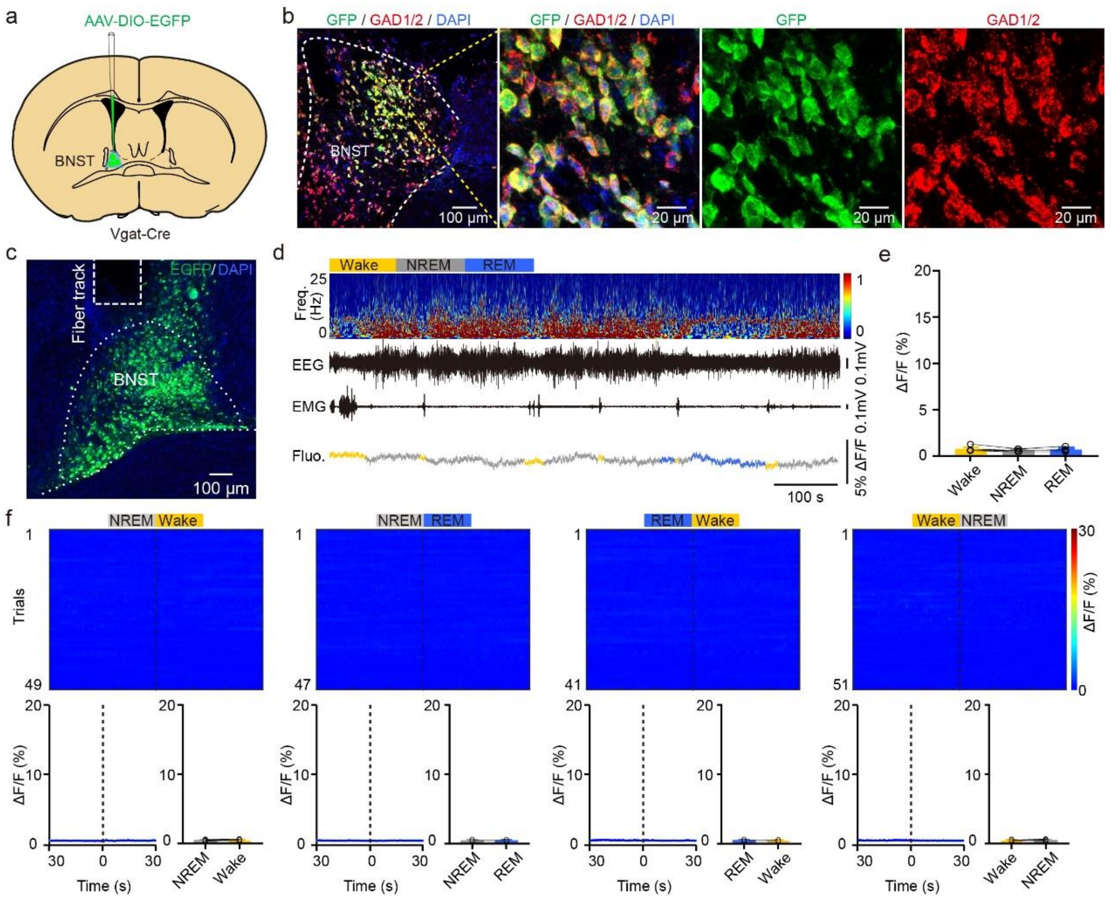

Supplementary Fig. 1 Fluorescence changes of EGFP-labeled BNST GABAergic neurons during natural sleep-wakefulness states. a Diagram of injection of AAV-DIO-EGFP into the BNST of *Vgat-Cre* mice. **b** Sections were co-stained with riboprobes for EGFP (green) and Gad1/2 (red). **c** Image illustrating AAV-DIO-EGFP expression (green) and the fiber tip location in the BNST with DAPI (blue) as the counterstain. **d** Representative EEG power spectrum, fluorescence changes, and EMG traces during natural sleep–wakefulness states. Yellow indicates wakefulness, grey indicates NREM sleep, and blue indicates REM sleep. Freq.: frequency. Fluo.: fluorescence **e** Quantification of fluorescence changes during wakefulness, NREM sleep, and REM sleep. *n* = 4 mice. **f** Top: heatmaps depicting individual recording traces aligned to sleep–wakefulness state transitions. NREM sleep to wake (49 transitions from 4 mice), NREM sleep to REM sleep (47 transitions from 4 mice), REM sleep to wake (41 transitions from 4 mice), and wake to NREM sleep (51 transitions from 4 mice). Bottom: average of all recording traces and statistical analysis from 30 s before and 30 s after state transitions. All transitions expressed as mean (blue) ± SEM (shaded). *n* = 4 mice. Data are presented as mean ± SEM. Statistical comparisons were determined using one-way RMs ANOVA with Bonferroni *post hoc* test or paired Student's *t*-test.

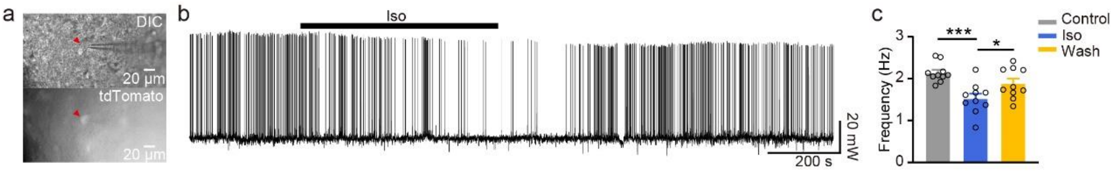

Supplementary Fig. 2 Isoflurane reduces the firing rate of BNST GABAergic neurons. a Visualization of recording BNST$^{Vgat\text{-}tdTomato}$ neurons. DIC: infrared-differential interference contrast. **b** The representative trace of BNST$^{Vgat\text{-}tdTomato}$ neurons under bath application of isoflurane. **c** Quantification of firing rates in BNST$^{Vgat\text{-}tdTomato}$ neurons under bath application of isoflurane. $n = 10$ neurons from 4 mice. Statistical comparisons were determined using one-way RMs ANOVA with Bonferroni *post hoc* test. $^{*}P < 0.05$, $^{***}P < 0.001$.

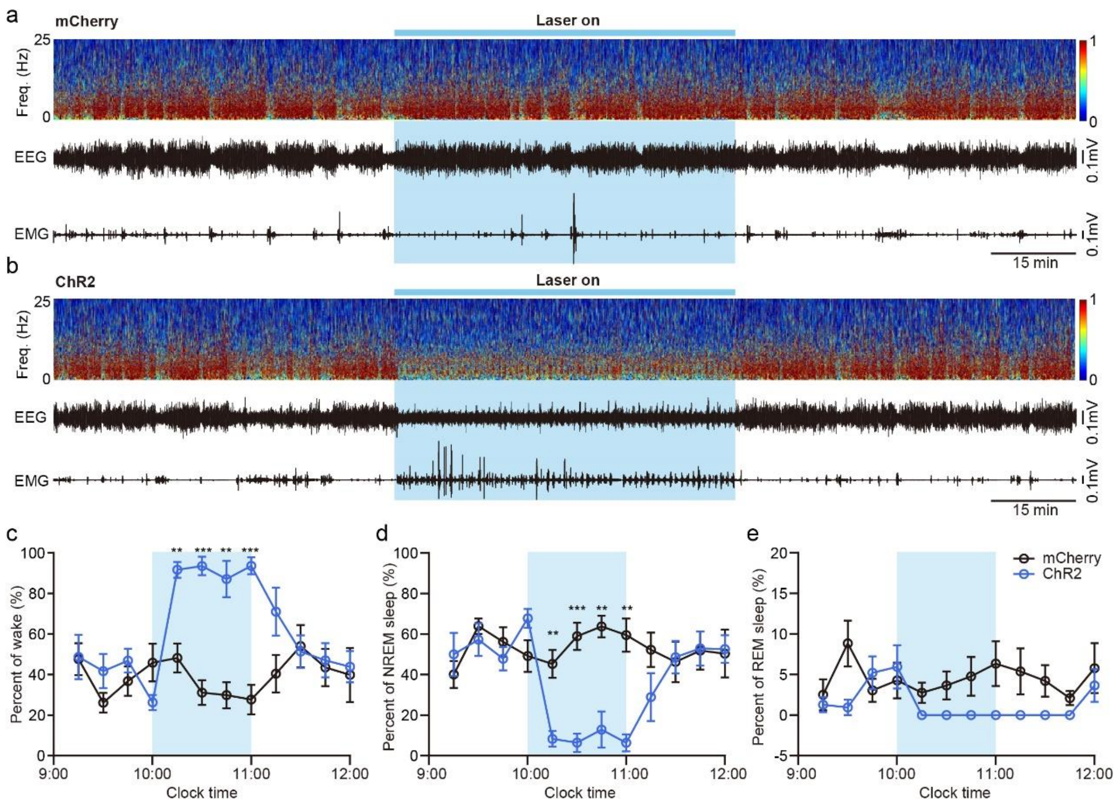

Supplementary Fig. 3 Prolonged optical stimulation of BNST GABAergic neurons maintains wakefulness. a-b Example of EEG power spectrum (top), raw EEG signals (middle), and EMG signals (bottom) during1 hour light stimulation in each group. Freq.: frequency. **c-e** Time spent in wakefulness **(c)**, NREM sleep **(d)**, and REM sleep**(e)** during prolonged-optogenetic activation experiments. Blue bands, 473nm laser stimulation (10 ms of 473 nm pulses at 20 Hz, 20 s on / 40 s off for 60 cycles). *n* = 8 mice in each group. Data are presented as mean ± SEM. Statistical comparisons were determined using two-way RMs ANOVA with Bonferroni *post hoc* test. ***P* < 0.01, ****P* < 0.001.

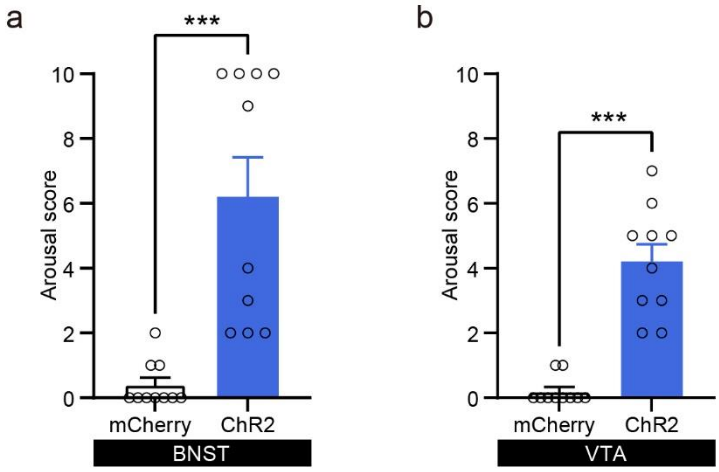

Supplementary Fig. 4 Arousal scores of *Vgat-Cre* mice following photostimulation of BNST GABAergic neurons or BNST GABAergic axonal terminals in the VTA during steady-state anesthesia. a Arousal scores based on behavioral responses of mice during steady-state anesthesia following photostimulation of BNST GABAergic neurons. $n = 10$ trials from 5 mice. **b** Arousal scores based on behavioral responses of mice during steady-state anesthesia following photostimulation of BNST GABAergic axon terminals in the VTA. $n = 10$ trials from 5 mice. Data are presented as mean ± SEM. Statistical comparisons were determined using Wilcoxon rank-sum test, ***$P < 0.001$.

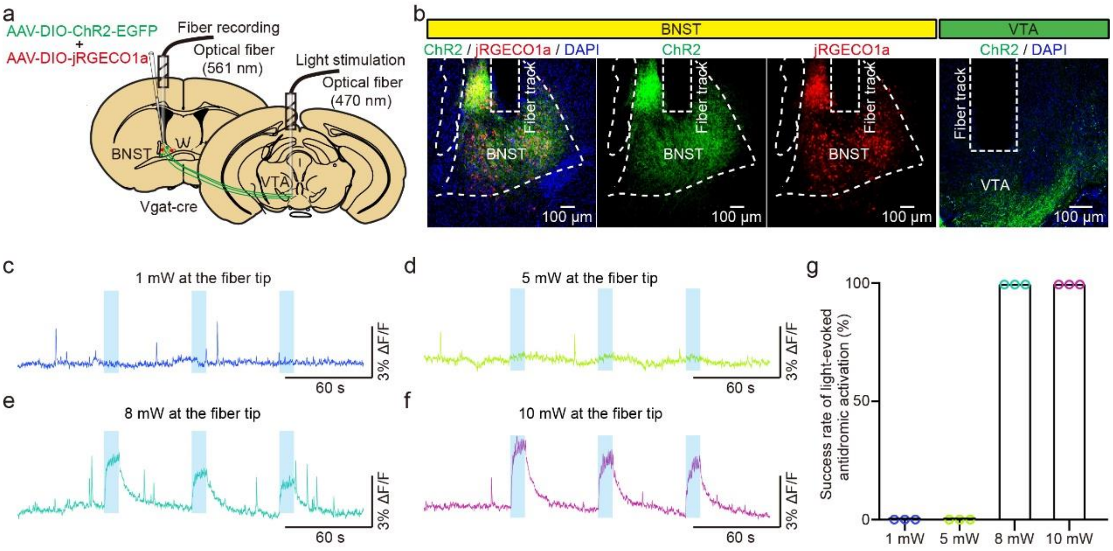

Supplementary Fig. 5 Activation of BNST GABAergic axon terminals in the VTA with light stimulation of 5 mW or less does not induce antidromic activation. a Diagram showing the injection of AAV-DIO-ChR2-EGFP and AAV-DIO-jRGECO1a into the BNST of *Vgat-Cre* mice, and fiber implantation for Ca^{2+} recording in BNST and for optogenetic stimulation in VTA. **b** Confocal images illustrating ChR2- (green) and jRGECO1a- (red) labeled somas in BNST, and ChR2-labeled axons in VTA. **c-f** Representative Ca^{2+} signals recorded from BNST GABAergic neurons with 1 mW **(c)**, 5 mW **(d)**, 8 mW **(e)**, and 10 mW **(f)** light stimulation at axonal terminals in the VTA. **g** Summary of the success rate for light-evoked antidromic activation in the BNST with VTA stimulation at 1 mW, 5 mW, 8 mW, and 10 mW. *n* = 3 mice.

	Leg movement	Head movement	Whisker movement	Righting	Walking	Total score
ChR2	0	0	2	0	0	2
ChR2	0	0	2	0	0	2
ChR2	2	2	2	2	2	10
ChR2	0	0	2	0	0	2
ChR2	1	1	2	0	0	4
ChR2	0	1	2	0	0	3
ChR2	2	2	2	2	2	10
ChR2	2	2	2	2	2	10
ChR2	2	2	2	2	2	10
ChR2	2	2	2	2	1	9
mCherry	0	0	1	0	0	1
mCherry	0	0	0	0	0	0
mCherry	1	0	0	0	0	1
mCherry	0	0	0	0	0	0
mCherry	0	0	0	0	0	0
mCherry	0	0	0	0	0	0
mCherry	0	0	0	0	0	0
mCherry	0	0	0	0	0	0
mCherry	1	1	0	0	0	2
mCherry	0	0	0	0	0	0

Supplementary Table 1. Behavioral responses of *Vgat-Cre* mice following photostimulation of BNST GABAergic neurons during steady-state anesthesia, related to Supplementary Fig. 4a. Behavioral responses were assessed following a 60 s photostimulation of BNST GABAergic neurons under steady-state anesthesia. Leg movement, head movement, whisker movement, states of the righting reflex and walking were evaluated for each animal. The intensity of each movement was scored on a scale of 0 (absent), 1 (mild), or 2 (moderate), depending on the strength of the observed motion. The total score is the sum of all categories, and the maximum score is 10.

	Leg movement	Head movement	Whisker movement	Righting	Walking	Total score
ChR2	2	2	2	0	0	6
ChR2	0	2	0	0	0	2
ChR2	1	2	2	0	0	5
ChR2	2	2	2	0	1	7
ChR2	1	2	1	0	0	4
ChR2	1	2	2	0	0	5
ChR2	0	2	1	0	0	3
ChR2	1	2	0	0	0	3
ChR2	1	2	2	0	0	5
ChR2	0	2	0	0	0	2
mCherry	0	0	0	0	0	0
mCherry	0	0	0	0	0	0
mCherry	0	0	0	0	0	0
mCherry	0	0	1	0	0	1
mCherry	0	0	0	0	0	0
mCherry	0	0	0	0	0	0
mCherry	0	0	0	0	0	0
mCherry	0	0	1	0	0	1
mCherry	0	0	0	0	0	0
mCherry	0	0	0	0	0	0

Supplementary Table 2. Behavioral responses of *Vgat-Cre* mice following photostimulation of BNST GABAergic axonal terminals in the VTA during steady-state anesthesia, related to Supplementary Fig. 4b. Behavioral responses were assessed following a 60 s photostimulation of BNST GABAergic axon terminals in the VTA under steady-state anesthesia. Leg movement, head movement, whisker movement, states of the righting reflex and walking were evaluated for each animal. The intensity of each movement was scored on a scale of 0 (absent), 1 (mild), or 2 (moderate), depending on the strength of the observed motion. The total score is the sum of all categories, and the maximum score is 10.
