# Supplementary material for: BNST GABAergic neurons modulate wakefulness over sleep and anesthesia
Source: Commun Biol. 2024 Mar 19;7:339. doi: 10.1038/s42003-024-06028-5 (PMC10950862; doi:10.1038/s42003-024-06028-5)
Supplement: Supplementary file 3 — Description of additional supplementary files [file 42003_2024_6028_MOESM3_ESM.docx]

Description of Additional Supplementary Files

**File name:** Supplementary data 1

**Description:** The source data behind the graphs in the paper
