# Supplementary material for: BNST GABAergic neurons modulate wakefulness over sleep and anesthesia
Source: Commun Biol. 2024 Mar 19;7:339. doi: 10.1038/s42003-024-06028-5 (PMC10950862; doi:10.1038/s42003-024-06028-5)
Supplement: Supplementary file 5 — Reporting Summary [file 42003_2024_6028_MOESM5_ESM.pdf]

nature portfolio

Corresponding author(s): Jingyu Xiao
Last updated by author(s): Feb 23, 2024

Reporting Summary

Nature Portfolio wishes to improve the reproducibility of the work that we publish. This form provides structure for consistency and transparency in reporting. For further information on Nature Portfolio policies, see our Editorial Policies and the Editorial Policy Checklist.

Statistics

For all statistical analyses, confirm that the following items are present in the figure legend, table legend, main text, or Methods section.

n/a	Confirmed	
☐	☒	The exact sample size (*n*) for each experimental group/condition, given as a discrete number and unit of measurement
☐	☒	A statement on whether measurements were taken from distinct samples or whether the same sample was measured repeatedly
☐	☒	The statistical test(s) used AND whether they are one- or two-sided<br>*Only common tests should be described solely by name; describe more complex techniques in the Methods section.*
☒	☐	A description of all covariates tested
☐	☒	A description of any assumptions or corrections, such as tests of normality and adjustment for multiple comparisons
☐	☒	A full description of the statistical parameters including central tendency (e.g. means) or other basic estimates (e.g. regression coefficient) AND variation (e.g. standard deviation) or associated estimates of uncertainty (e.g. confidence intervals)
☐	☒	For null hypothesis testing, the test statistic (e.g. *F*, *t*, *r*) with confidence intervals, effect sizes, degrees of freedom and *P* value noted<br>*Give P values as exact values whenever suitable.*
☒	☐	For Bayesian analysis, information on the choice of priors and Markov chain Monte Carlo settings
☒	☐	For hierarchical and complex designs, identification of the appropriate level for tests and full reporting of outcomes
☒	☐	Estimates of effect sizes (e.g. Cohen's *d*, Pearson's *r*), indicating how they were calculated

Our web collection on statistics for biologists contains articles on many of the points above.

Software and code

Policy information about availability of computer code

Data collection	The calcium signals were digitized using customized acquisition LabVIEW software (National instrument).
Data analysis	Fiber recordings were first processed by LabVIEW software (National Instruments) and then analyzed by Igor Pro 5.0 (Wavemetrics). The raw EEG data were band-pass filtered (0-25 Hz) and calculated by fast-Fourier transformation with a frequency resolution of 0.2 Hz through SleepSign for animals (Kissei Comtec, Japan). To quantify burst suppression, raw EEG data were converted to text format for further analysis of BSR using a Hilbert transform by MATLAB R2020a software. Statistical analyses were conducted using Graph Pad Prism 9.0 (GraphPad Software, USA) and SPSS 27.0 software (IBM SPSS Statistics for Windows, USA).

For manuscripts utilizing custom algorithms or software that are central to the research but not yet described in published literature, software must be made available to editors and reviewers. We strongly encourage code deposition in a community repository (e.g. GitHub). See the Nature Portfolio guidelines for submitting code & software for further information.

Data

Policy information about availability of data

All manuscripts must include a data availability statement. This statement should provide the following information, where applicable:

- Accession codes, unique identifiers, or web links for publicly available datasets
- A description of any restrictions on data availability
- For clinical datasets or third party data, please ensure that the statement adheres to our policy

The data that support the findings of this study are available from the corresponding author upon reasonable request.

Research involving human participants, their data, or biological material

Policy information about studies with human participants or human data. See also policy information about sex, gender (identity/presentation), and sexual orientation and race, ethnicity and racism.

Reporting on sex and gender	N/A
Reporting on race, ethnicity, or other socially relevant groupings	N/A
Population characteristics	N/A
Recruitment	N/A
Ethics oversight	N/A

Note that full information on the approval of the study protocol must also be provided in the manuscript.

Field-specific reporting

Please select the one below that is the best fit for your research. If you are not sure, read the appropriate sections before making your selection.

☒ Life sciences ☐ Behavioural & social sciences ☐ Ecological, evolutionary & environmental sciences

For a reference copy of the document with all sections, see nature.com/documents/nr-reporting-summary-flat.pdf

Life sciences study design

All studies must disclose on these points even when the disclosure is negative.

Sample size	Sample sizes were determined based on similar experiments using in vivo fiber photometry and optogenetic methods. Samples sizes adopted in this study were sufficient for detecting robust effect.
Data exclusions	Mice of those virus injection or fiber tip was not in the target area were excluded from experimental analysis.
Replication	For all experiments, replications were conducted successfully. We describe detailed methods and sources of all reagents and protocols for experiments, in order to ensure that experimental findings can be reliably reproduced.
Randomization	Mice were randomly assigned to experimental and control groups.
Blinding	All data were analyzed by investigators who were blind to the conditions of the mice and each experiment.

Reporting for specific materials, systems and methods

We require information from authors about some types of materials, experimental systems and methods used in many studies. Here, indicate whether each material, system or method listed is relevant to your study. If you are not sure if a list item applies to your research, read the appropriate section before selecting a response.

Materials & experimental systems

n/a	Involved in the study
☐	☒ Antibodies
☒	☐ Eukaryotic cell lines
☒	☐ Palaeontology and archaeology
☐	☒ Animals and other organisms
☒	☐ Clinical data
☒	☐ Dual use research of concern
☒	☐ Plants

Methods

n/a	Involved in the study
☒	☐ ChIP-seq
☒	☐ Flow cytometry
☒	☐ MRI-based neuroimaging

Antibodies

Antibodies used: rabbit anti-NeuN 1:400, ab177487, Abcam, RRID: AB_2532109

Validation: The application and specificity of all the antibodies are validated by the companies.

Animals and other research organisms

Policy information about studies involving animals; ARRIVE guidelines recommended for reporting animal research, and Sex and Gender in Research

Laboratory animals: Vgat-Cre mice were obtained from Jackson Laboratory. Vgat-Cre mice were crossed with Cre-dependent tdTomato reporter knock-in mice (B-tdTomato cKI mice, Beijing Biocytogen Inc.). 8 to 20-week-old mice of both sexes were utilized for the experiment. Mice at least 11 weeks old were used for recording and manipulation experiments.

Wild animals: The study did not use wild animals.

Reporting on sex: Male and female mice were both used in the experiment.

Field-collected samples: N/A

Ethics oversight: All experimental procedures were approved by the Third Military Medical University Animal Care and Use Committee and experiments were performed according to institutional animal welfare guidelines.

Note that full information on the approval of the study protocol must also be provided in the manuscript.

Plants

Seed stocks: N/A

Novel plant genotypes: N/A

Authentication: N/A
